# Effect of New Zealand Blackcurrant Supplementation on Sports Performance: A Systematic Review

**DOI:** 10.1016/j.cdnut.2026.109388

**Published:** 2026-06-02

**Authors:** Samira Gholamian, Sina Ebrahimi, Arezoo Razmdideh, Mohammad Shariatzadeh, Ehsan Rezaee Moeini

**Affiliations:** 1 Sport Sciences Research Institute, Tehran, Iran; 2 Department of Exercise Physiology, Faculty of Physical Education and Sport Sciences, Islamic Azad University, Sanandaj Branch, Sanandaj, Iran; 3 Department of Physical Education and Sport Sciences, Faculty of Literature, Humanities and Social Sciences, Science and Research Branch, Islamic Azad University, Tehran, Iran; 4 Sport Sciences Research Institute, Tehran, Iran; 5 Department of Exercise Physiology, Sport Sciences Research Institute, Tehran, Iran

**Keywords:** athletic performance, exercise training, fat oxidation, polyphenols, anthocyanin

## Abstract

**Background:**

New Zealand blackcurrant (NZBC) is a rich source of anthocyanins, but findings on its effects on sports performance and metabolic health are inconsistent.

**Objectives:**

This systematic review aimed to evaluate the effects of NZBC supplementation on sport performance and metabolic outcomes in adults.

**Methods:**

A comprehensive literature search was conducted to identify randomized crossover clinical trials assessing NZBC supplementation. Standardized effect sizes (Cohen’s *d*) were extracted where sufficient data were available. Random-effects models were applied, and subgroup analyses were conducted according to sport type.

**Results:**

Twenty-one randomized crossover clinical trials were included. Overall, NZBC supplementation had a small-to-moderate effect on sport performance (mean *d* = 0.44). Sport-specific analyses showed the largest effects in intermittent and team-based activities involving repeated high-intensity efforts (*d* = 0.6). Effects in endurance cycling were smaller and variable, and absent under hypoxic conditions. Continuous running, walking, and neuromuscular tasks showed small or inconsistent effects. Metabolic outcomes, particularly fat oxidation, showed more consistent improvements. Although most trials had low risk of bias at the individual study level, the certainty of evidence was low for sport performance outcomes and moderate for metabolic outcomes due to heterogeneity, imprecision, and small sample sizes.

**Conclusions:**

NZBC supplementation may provide modest benefits for sports performance and metabolic outcomes. However, variability across doses, exercise modalities, and study designs highlights the need for larger, well-controlled trials to clarify its efficacy.

## Introduction

Physical exercise has consistently demonstrated significant benefits for cardiovascular disease, cancer, metabolic, and musculoskeletal disorders, as well as overall physical and mental health [[1], [2], [3], [4], [5], [6], [7], [8], [9]].

Recent evidence suggests that the health benefits of plants are largely attributed to their bioactive compounds, namely polyphenols, terpenoids, and alkaloids, which exert diverse physiological effects [[10], [11], [12], [13]]. Blackcurrant (*Ribes nigrum*), a dark purple berry native to Europe and Asia, is among the richest natural sources of polyphenols, particularly anthocyanins such as delphinidin-3-rutinoside, delphinidin-3-glucoside, cyanidin-3-rutinoside, and cyanidin-3-glucoside. New Zealand blackcurrants (NZBC) are a specific cultivar grown under intense UV light, extended daylight hours, and unique soil conditions, resulting in exceptionally high levels of anthocyanins [11,[13], [14], [15], [16]].

NZBC has gained increasing attention as a sports nutritional supplement with the potential to enhance performance by increasing blood vessel diameter [13,17,18], blood flow [18,19], and regulating lipid metabolism [13,20,21]. Furthermore, studies suggest that NZBC promotes fat oxidation during endurance exercise and reduces carbohydrate oxidation, leading to a decrease in respiratory exchange ratio. This metabolic shift toward fat utilization is not only advantageous for endurance performance, but may also improve lipid profiles with chronic intake [13,22,23]. However, findings regarding the effects of NZBC on sports performance remain conflicting. For instance, some studies have reported improvements in cycling time trials [24], running [25], and climbing [26]; whereas others have observed no effect of NZBC on sports performance [27,28].

Since the publication of the 2020 systematic review [20], a significant number of additional studies have investigated the effects of NZBC supplementation on exercise performance and metabolic outcomes. However, these newer studies vary widely in terms of exercise modality, dosing strategy, and duration of supplementation, and their findings have not yet been synthesized. Therefore, an updated systematic review is warranted to incorporate this emerging evidence and specifically examine potential dose-related effects and exercise-type-specific responses, which are not yet well understood. Addressing these gaps is essential to clarify the ergogenic potential of NZBC supplements and provide evidence-based recommendations for athletes and physically active individuals.

## Methods

The PRISMA statement, published in 2020, was used as a guideline for this study. The protocol for this review was not registered with any organization. All the steps, including literature searching, study selection, and quality assessment, were independently conducted by 2 authors, with any discrepancies resolved through discussion or consultation with a third reviewer. Ethics approval is not required for the analysis of published articles.

### Search strategy

A comprehensive literature search was conducted across PubMed, Embase, SPORTDiscus (EBSCOhost), and Cochrane CENTRAL from January 2005 to July 2025. The search was restricted to English-language studies involving human participants. The detailed search strategy is provided in Appendix 1.

### Inclusion and exclusion criteria

Randomized controlled trials (RCTs) involving healthy, physically active individuals (athletes or recreationally active) that evaluated the effects of NZBC supplementation compared with a control on sport or exercise performance outcomes were included. Observational studies (case series and case reports), animal and in vitro experiments, studies in clinical populations (e.g., metabolic syndrome, cardiovascular disease, and autoimmune disorders), trials combining NZBC with other supplements, non-English publications, reviews, and conference abstracts were excluded. Grey literature, including theses, dissertations, and unpublished studies, was not considered. Only peer-reviewed articles were included. Case studies are mentioned contextually and are not included in the composition.

### Study selection

All records identified through database searches were imported into EndNote reference management software, and duplicates were removed. Titles and abstracts were independently screened by 2 reviewers against the eligibility criteria, followed by full-text assessment of potentially relevant studies.

### Data extraction

Data were independently extracted by 2 reviewers using a standardized form, including study and participant characteristics, intervention details (form, dose, and duration), comparators, and outcomes. Primary outcomes were sports and exercise performance measures, whereas secondary outcomes included lipid profile parameters and metabolic responses. To avoid unit-of-analysis errors and double-counting, each study contributed only one independent effect size per outcome category to the quantitative synthesis.

### Risk of bias assessment

The Risk of Bias 2 (RoB 2) tool is used to assess the risk of bias in individual randomized trials. It evaluates the methodological quality across 5 domains: randomization, deviation from intended interventions, missing outcome data, outcome measurement, and selection of reported outcomes. For RCTs and crossover studies, the Cochrane RoB 2 tool was applied. The latest version of the RoB 2 includes an extension for crossover trials, accounting for design-specific aspects such as carry-over effects, period effects, and sequence generation. Each study was categorized as having low, moderate, or high risk of bias (or good, moderate, or poor quality) according to the relevant tool.

### Grading of evidence (Grading of Recommendations Assessment, Development, and Evaluation)

The certainty of evidence for each outcome was assessed using the Grading of Recommendations Assessment, Development, and Evaluation (GRADE) approach, which considers risk of bias, inconsistency of results, indirectness of evidence, imprecision of estimates, and potential publication bias. Risk of bias in individual studies was evaluated using the RoB 2 tool, and these judgements inform the GRADE assessment. The overall certainty of the evidence for each outcome (sports performance and fat oxidation) was classified as high, moderate, low, or very low.

### Analysis

Because most crossover trials did not report within-participant correlations, a conservative independent-groups variance approximation was applied, which may underestimate precision but avoids unit-of-analysis errors. Data were synthesized qualitatively, and where sufficient data were available, standardized effect sizes (Cohen’s *d*) were extracted, and random-effects models were applied. First, effect sizes for exercise performance (Cohen's *d*) were extracted directly when reported, or calculated from available means and SDs when not provided. For crossover studies lacking pairwise difference data, intervention and control conditions were treated as independent groups with equal sample sizes, in accordance with standard meta-analysis guidelines [[29], [30], [31]]. These Cohen’s *d* values were then entered into the random-effects model to estimate pooled effects. For pooling, small-sample correction was applied where appropriate (i.e., conversion to Hedges’ *g* for the meta-analytic step), but results were reported and interpreted in terms of Cohen’s *d* for consistency across tables and figures.

### Heterogenity

Because of the small number of studies per outcome and the predominance of crossover designs, formal heterogeneity statistics (e.g., *I*^2^) are not reported, as they may be underpowered and potentially misleading.

## Results

**FIGURE 1:**
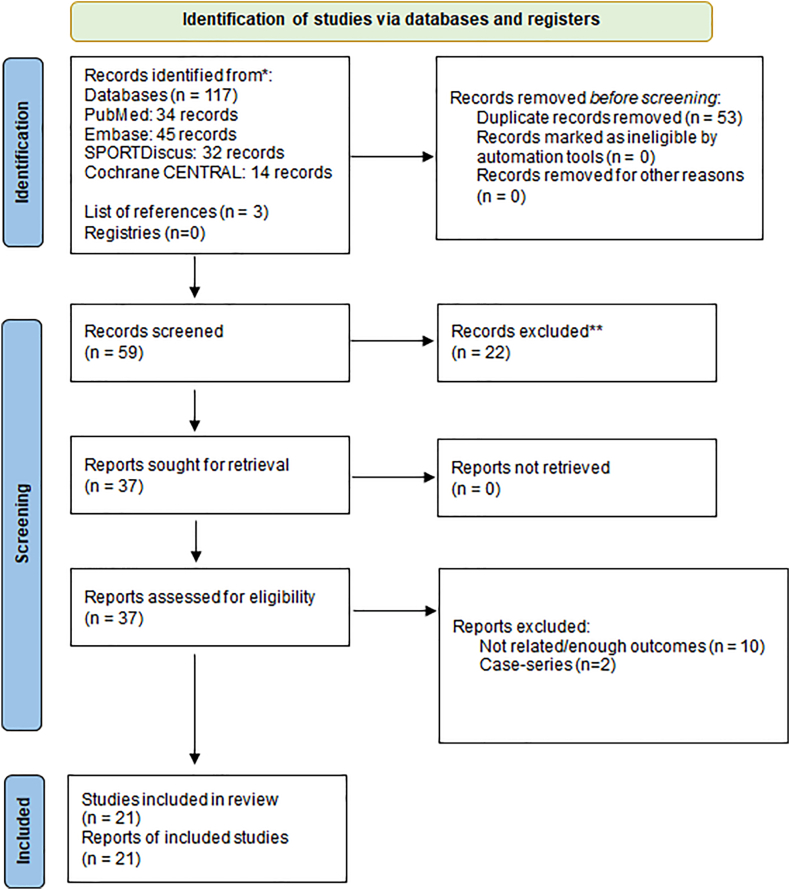
PRISMA flow diagram for the present systematic review.

Figure 1 shows the study selection process, and Table 1 summarizes the characteristics and extracted data of each included study. A total of 117 records were identified through database searches. After removing 53 duplicates, 59 records remained for screening. Of these, 22 were excluded based on title and abstract review, leaving 37 full-text articles assessed for eligibility. Among them, 16 articles were excluded (14 due to insufficient or unrelated outcomes, and 2 as case series [22,23]). Finally, 21 studies met the inclusion criteria and were included in the systematic review [[24], [25], [26], [27], [28],[32], [33], [34], [35], [36], [37], [38], [39], [40], [41], [42], [43], [44], [45], [46], [47], [48], [49], [50], [51], [52]].

**TABLE 1:** Summary of extracted data on effect of NZBC on sport performance

Author (y, country)	Participants	Study design	Intervention (form, dose, duration)	Comparator, exercise test	Effect size for sport performance (Cohen’s *d)*	*P* value	Direction of effect	Main outcomes (sport performance/metabolism)
Burnett and Willems, 2022 (United Kingdom) [32]	13 (21 ± 2 y)	RCT, crossover	210 mg/d anthocyanins × 7 d	Rugby (6 × 35 m sprints)	*d* ≈ 0.56 (moderate)	0.03	Favors NZBC	Sport performance/small-to-moderate sprint and agility benefits; no effect on strength
Cook, 2015 (United Kingdom) [24]	14 (38 ± 13 y)	RCT, crossover	105 mg/d anthocyanins × 7 d	Cycling (30 min submax + 16.1 km TT)	*d* ≈ 0.7 (moderate–large)	0.027	Favors NZBC	Metabolism / ↑ fat oxidation (+27%), ↑ TT performance (+2.4%)
Cook, 2022 (United Kingdom) [33]	13 (25 ± 4 y)	RCT, crossover	210 mg/d anthocyanins × 7 d	Isometric contraction	*d* ≈ 0.24 (small)	0.003	Favors NZBC	Time-specific effects after 7 d
Costello, 2020 (United Kingdom) [34]	20 (30 ± 6 y)	RCT	105 mg/d anthocyanins × 7 d	Running (half-marathon)	NR	>0.05	Null	↑ inflammation = worse Negative/unfavorable
Fryer, 2020 (United Kingdom) [35]	12 (elite climbers)	RCT, crossover	210 mg/d anthocyanins × 7 d	Climbing (forearm tests)	NR	>0.05	Favors NZBC for physiological benefits.	Sport performance: null Muscle oxidative capacity improved min-TSI % (oxygen extraction) improved
Fryer, 2021 (United Kingdom) [36]	12 (elite climbers)	RCT, crossover	210 mg/d anthocyanins × 7 d	Climbing (forearm tests)	NR	<0.001	Favors NZBC for physiological benefits only	Sport performance: null Improvements in muscle oxidative capacity improved, min-TSI %
Godwin, 2017 (United Kingdom) [37]	24 (youth footballers, 17–20 y)	RCT, crossover	210 mg/d anthocyanins × 7 d	Football (6 × 35 m sprints)	*d* ≈ 0.89 (large)	0.02	Favors NZBC	Improved repeated sprint in trained players only
Montanari, 2020 (United Kingdom) [27]	13 (cyclists)	RCT, crossover	105 mg/d anthocyanins × 7 d	Cycling TT	Null	>0.05	Null	Small improvement on day 4 with 600 mg; otherwise null
Montanari, 2023 (United Kingdom) [38]	34 (26 M, 8 F; 38 ± 7 y)	RCT, crossover	315 mg/d anthocyanins × 7 d (2 h pre-TT)	16.1 km cycling TT	*d* ≈ 0.23 for slower cyclists only	0.02	Favors NZBC	Improvement in slower cyclists only; no overall effect for all cyclists
Morton, 2025 (United Kingdom) [28]	16 (37 ± 11 y)	RCT, crossover	315 mg/d anthocyanins × 7 d (2 h pre-TT)	Cycling	Null	>0.05	Null	No significant effects on performance or metabolism
Moss, 2023 (United Kingdom) [39]	16 (26 ± 5 y)	RCT, crossover	315 mg/d anthocyanins × 7 d (2 h pre-TT)	5 km running TT	*d* ≈ 0.23 (small, significant)	0.001	Favors NZBC	Faster time; no physiological differences
Murphy, 2017 (United Kingdom) [40]	10 (30 ± 12 y)	RCT, crossover	105 mg/d anthocyanins × 7 d	Cycling (2 × 4 km TT)	Null	>0.05	Null	0.8% faster total time; no metabolic effects
Naderi, 2025 (United Kingdom) [41]	9 (elite rowers)	RCT, crossover	210 mg/d anthocyanins × 7 d	2000-m rowing ergometer	Null	>0.05	Null	No statistically significant performance benefit
Perkins, 2015 (United Kingdom) [25]	13 (25 ± 4 y)	RCT, crossover	105 mg/d anthocyanins × 7 d	HIIR running	*d* ≈0.56 (moderate)	0.02	Favors NZBC	↑ Distance (+10.6%); ↑ sprint distance (+10.8%)
Perkins, 2024 (United Kingdom) [42]	16 (23 ± 3 y)	RCT, crossover	210 mg/d anthocyanins × 7 d	HIIR running	NR Responder rate: 50%	NR	Favors NZBC	↑ Distance (∼8%); ∼50% responders showed large gains No effect on mean heart rate, mean oxygen uptake ($\dot{V}$O_2_), mean blood lactate
Potter, 2020 (United Kingdom) [26]	18 (24 ± 6 y)	RCT, crossover	210 mg/d anthocyanins × 7 d	Climbing	NR	NR	Favors NZBC for climbing time only null overall	↑ Climbing time (+23%); no effect on pull-ups, HR, lactate, FG, HGS
Şahin, 2021 (United Kingdom) [43]	16 (24 ± 6 y)	RCT, crossover	210 mg/d anthocyanins × 7 d	Walking (30 min, moderate)	7 d: *d* ≈ 0.24 (small) 14-d: *d* ≈ 0.42 (small–moderate)	0.007	Favors NZBC	Improvements in fat oxidation, CHO oxidation, RER, RPE, no effect on HR/dose-duration dependent
Strauss, 2018 (United Kingdom) [44]	16 (28 ± 8 y)	RCT, crossover	210 mg/d anthocyanins × 7 d	Cycling (120 min at 65% VO_2_ max)	*d* ≈ 0.55 (moderate)	0.042	Favors NZBC	↑ Fat oxidation (+27%); ↓ carbohydrate oxidation (trend)
Willems, 2016 (United Kingdom) [45]	13 (22 ± 1 y)	RCT, crossover	105 mg/d anthocyanins × 7 d	LIST (intermittent running)	*d* ≈ 0.60 (moderate) for fastest sprint slowing only	<0.05	Favors NZBC	↓ Sprint slowing; no significant effect on heart rate, VO_2_, blood lactate, Vertical jump power, Avg sprint time
Willems, 2020 (United Kingdom) [46]	12 (24 ± 5 y)	RCT, crossover	210 mg/d anthocyanins × 7 d	Isometric contractions	NR	0.05	Favors NZBC	+12% improvement in baseline twitch force No effects on MVC force (late quartile), Twitch force (fatigue and recovery)
Willems, 2019 (United Kingdom) [47]	11 (38 ± 11 y)	RCT, crossover	210 mg/d anthocyanins × 7 d	6.1 km cycling TT	*d* ≈ 0.0	0.97	Null	No effect on TT, metabolism, or HR

A dose of 300 mg/d of NZBC extract was approximately equivalent to 105 mg of anthocyanins, 600 mg/d of NZBC extract was approximately equivalent to 210 mg of anthocyanins, and 900 mg of NZBC extract was approximately equivalent to 315 mg of anthocyanins in all included studies.

Abbreviations: CHO, carbohydrate; FG, forearm girth; HIIR, high-intensity intermittent running; HGS, handgrip strength; HR, heart rate; LIST: loughborough intermittent shuttle test; min-TSI: minimum tissue saturation index; MVC, maximal voluntary contraction; NR, not reported; Null, no effect of NZBC; NZBC, New Zealand blackcurrant; RCT, randomized controlled trial; RER, respiratory exchange ratio; RPE, rating of perceived exertion; TT, time trial; VO_2_: mean oxygen uptake.

All studies were RCT crossover designs. Of 21 included studies, 4 reported outcomes exclusively on metabolism and/or physiology (i.e., no direct sport performance outcome) [35,36,42,48], and 17 studies reported outcomes on both metabolism and sport performance [[23], [24], [25], [26], [27], [28],[32], [33], [34],[37], [38], [39], [40], [41], [42], [43],^45^,^46^,^47^,^49^]. Six studies reported the use of 105 mg of anthocyanins per day [24,25,27,33,40,45, 12[24,25,27,33,40,45], 12 studies reported the use of 210 mg of anthocyanins per day [ 26,32,33,35,36,37,41-44,46,47], and 3 studies reported the use of 315 mg of anthocyanins per day [28,38,39]. Table 1 presents a summary of the characteristics and extracted data.

### Analysis

#### Descriptive overall effect size

**FIGURE 2:**
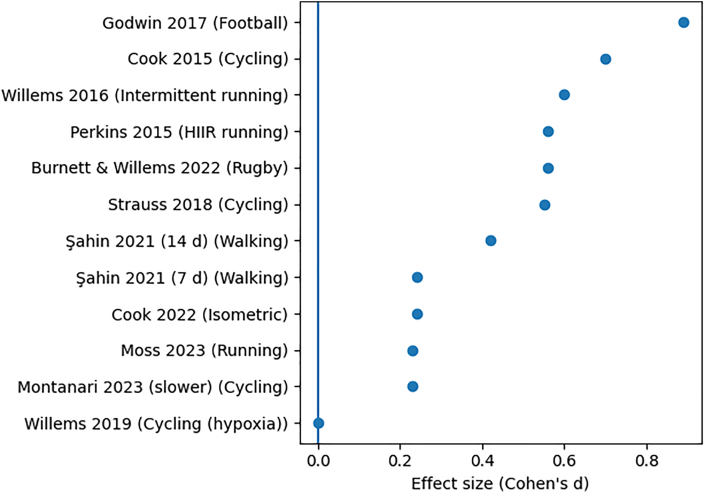
Forest plot of NZBC effect sizes on sport performance, grouped by sport type, using only studies that reported a numeric Cohen’s *d* (as is appropriate for a quantitative synthesis). HIIR, high-intensity intermittent running; NZBC, New Zealand blackcurrant.

Figure 2 presents the forest plot of NZBC supplementation standardized effect sizes (Cohen’s *d*), on sports performance according to the type of sport. The descriptive overall effect size of NZBC extract on sport performance was small to moderate, with a mean unweighted effect size of approximately *d* = 0.44 and a median effect size of *d* = 0.49. These values indicate a generally positive direction of effect in favor of NZBC supplementation across diverse sport performance outcomes.

#### Random-effects pooled estimates

In the absence of reported within-subject correlations, a standard independent-groups approximation for the variance of Cohen’s *d* was used to calculate random-effects pooled estimates, consistent with established meta-analytic guidance [30,31]. Table 2 presents calculated random-effects pooled estimates based on Cohen’s *d* by sport category. Cycling shows a small-to-moderate pooled effect, but is context-dependent (includes a hypoxia null and a slower-cyclist subgroup effect). Walking shows a small pooled effect (but this is more “metabolic/perceptual” than sport performance). The largest pooled performance effect is in intermittent/team sports (moderate).

**TABLE 2:** Random-effects pooled estimates by sport category (Cohen’s *d*)

Sport category	k	Pooled *d* (RE)	95% CI
Intermittent/team (sprints and HIIR)	4	0.66	0.34, 0.98
Cycling	4	0.38	0.01, 0.75
Walking/moderate exercise	2	0.33	−0.16, 0.82
Continuous running	1	0.23	−0.47, 0.93
Isometric/neuromuscular	1	0.24	−0.53, 1.01

Categories with *k* = 1 are just the single-study estimate (not a true pooled effect).

Abbreviations: CI, confidence interval; HIIR, high-intensity intermittent running; RE, random effects.

### Publication bias

**FIGURE 3:**
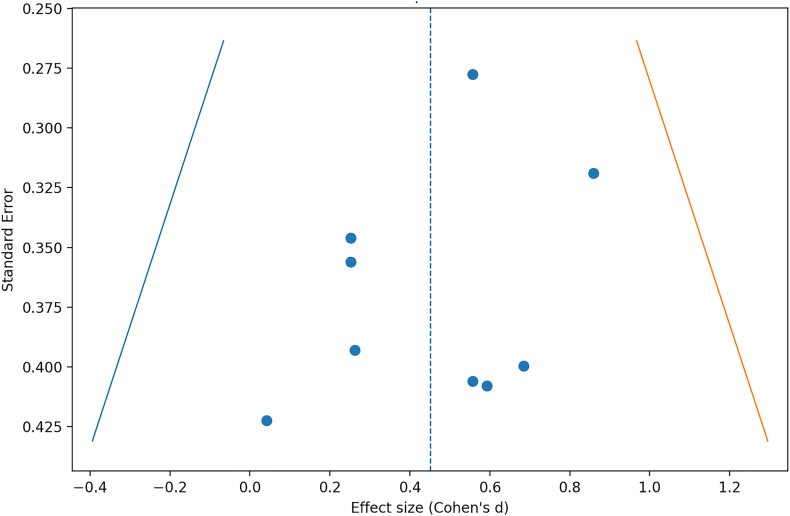
The funnel plot for the 9 studies reporting Cohen’s *d* on sport performance. A vertical dashed line shows the pooled/mean effect (symmetry axis). The 2 diagonal lines show the 95% pseudoconfidence limits.

Potential publication bias was explored using funnel plots only for studies with a numeric effect size (Cohen’s *d*) [24,25,32,33,[37], [38], [39],^45^,^47^] (Figure 3). Visual inspection of the funnel plot based on 9 studies reporting standardized sport performance effect sizes did not indicate marked asymmetry; however, given the small number of studies contributing to each pooled analysis, these assessments were considered exploratory and interpreted with caution.

### Risk of bias assessment

**FIGURE 4:**
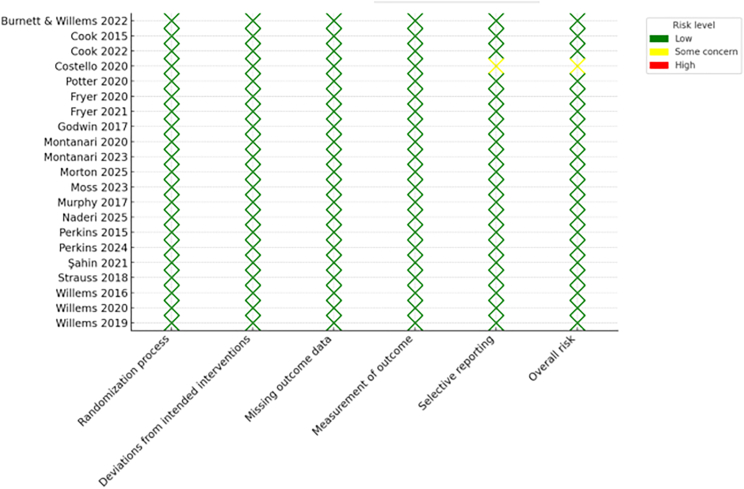
Risk of bias summary.

Table 3 and Figure 4 present the results of the risk of bias assessment. Overall, the methodological quality of the included RCTs was considered good. Most studies were judged to have a low risk of bias across all domains, including randomization, deviations from intended interventions, missing outcome data, and outcome measurement. Only 1 study (Costello 2020) raised some concern due to selective reporting (IL-6 outcomes reported only in the NZBC group). No studies were rated as having a high risk of bias. Overall, the evidence base for NZBC supplementation and exercise performance outcomes appears methodologically robust with minimal concerns regarding bias.

**TABLE 3:** Risk of bias structured table using Cochrane RoB 2 domains

Study	Randomization process	Deviations from intended interventions	Missing outcome data	Measurement of outcome	Selective reporting	Overall risk
Burnett and Willems 2022 [32]	Low	Low	Low	Low	Low	Low
Cook 2015 [24]	Low	Low	Low	Low	Low	Low
Cook 2022 [33]	Low	Low	Low	Low	Low	Low
Costello 2020 [34]	Low	Low	Low	Low	Some concern (IL-6 only in NZBC)	Some concern
Potter 2020 [26]	Low	Low	Low	Low	Low	Low
Fryer 2020 [35]	Low	Low	Low	Low	Low	Low
Fryer 2021 [36]	Low	Low	Low	Low	Low	Low
Godwin 2017 [37]	Low	Low	Low	Low	Low	Low
Montanari 2020 [27]	Low	Low	Low	Low	Low	Low
Montanari 2023 [38]	Low	Low	Low	Low	Low	Low
Morton 2025 [28]	Low	Low	Low	Low	Low	Low
Moss 2023 [39]	Low	Low	Low	Low	Low	Low
Murphy 2017 [40]	Low	Low	Low	Low	Low	Low
Naderi 2025 [41]	Low	Low	Low	Low	Low	Low
Perkins 2015 [25]	Low	Low	Low	Low	Low	Low
Perkins 2024 [42]	Low	Low	Low	Low	Low	Low
Şahin 2021 [43]	Low	Low	Low	Low	Low	Low
Strauss 2018 [44]	Low	Low	Low	Low	Low	Low
Willems 2016 [45]	Low	Low	Low	Low	Low	Low
Willems 2020 [46]	Low	Low	Low	Low	Low	Low
Willems 2019 [47]	Low	Low	Low	Low	Low	Low

Abbreviations: NZBC, New Zealand blackcurrant; RoB 2, Risk of Bias 2.

### GRADE assessment

Using the GRADE assessment tool, the certainty of evidence was rated as low for exercise performance due to inconsistency and imprecision across small crossover trials. In contrast, the certainty of evidence for metabolic outcomes was rated as moderate, reflecting more consistent findings indicating enhanced fat oxidation and altered substrate utilization following NZBC supplementation (Table 4).

**TABLE 4:** Summary of findings (GRADE)

Outcome	Number of studies	Study design	Certainty of evidence
Exercise performance	17 RCTs	Randomized crossover	Low ◯◯◯◯
Metabolism	8 RCTs	Randomized crossover	Moderate ●●◯◯

Abbreviations: GRADE, Grading of Recommendations Assessment, Development, and Evaluation; RCT, randomized controlled trial.

## Discussion

A previous study including 9 studies indicated that NZBC at doses of 105–210 mg total anthocyanins per day may improve sports performance [20]. However, the present updated systematic review indicates that the ergogenic effects of NZBC supplementation are exercise-specific and dose-dependent, rather than uniform across sports or dosing strategies. The descriptive overall effect was small to moderate (*d* = 0.44), indicating a small-to-moderate median improvement in sport performance with NZBC supplementation compared with control. Random-effects pooling showed the most consistent and largest performance benefits in intermittent and team-based activities characterized by repeated high-intensity efforts and cumulative fatigue. Although most included trials were assessed as having a low risk of bias at the individual study level, the certainty of evidence at the outcome level remains low for exercise performance outcomes and moderate for metabolic outcomes, primarily due to heterogeneity in exercise modalities, imprecision, and small sample sizes inherent to crossover designs.

Climbing and running demonstrated moderate benefit, with several studies reporting improvements in climbing performance, muscle oxygenation, recovery, faster 5 km times, and performance in intermittent protocols [26,35,36]. Team-sport tasks, including rugby and football, also showed small but significant improvements in sprint and agility, particularly in trained athletes [32,37]. Evidence supporting muscle function, such as strength, stability, and isometric contractions, indicates moderate effects potentially reflecting the response in specific fibers [33,46]. Cycling results were less consistent, with some studies showing minor improvements in time-trial performance, whereas others reported no effect on performance or metabolism. Two separate clinical trials by Montanari et al. [38]—one using an acute high dose of NZBC extract and another employing a daily chronic dose—found limited and inconsistent benefits [27]. The acute high-dose study showed improvements only in slower cyclists, whereas the chronic 600 mg/d regimen produced small gains during days 1–4, but no consistent effect overall. Both studies suggest that the effects of NZBC on cycling time-trial performance may depend on baseline fitness, dose, duration, and possibly sex. Morton et al. [28] reported that neither acute nor 7-d supplementation significantly affected cycling performance, physiology, or metabolism. Conversely, Cook et al. [24] demonstrated that 1 wk of NZBC extract (300 mg/d; 105 mg anthocyanins) increased fat oxidation by 27% and improved 16.1-km time-trial performance by 2.4% compared with placebo (*P* < 0.05; *P* < 0.05).

Dose and duration appear to be important modifiers of response; however, the current evidence does not support a definitive inverse dose-response relationship. Rather than indicating that “moderate doses are most effective,” the data suggest that mid-range anthocyanin intakes (∼210–300 mg/d) are more frequently associated with performance and metabolic benefits across studies, whereas both lower and higher doses demonstrate greater variability and task-specific effects. Higher doses (600–900 mg extract; ∼210–315 mg anthocyanins) were beneficial in certain contexts, such as climbing, running, and suboptimal cycling capacity, but showed little or no benefit in rowing, some cycling time trials, and postmarathon recovery. These heterogeneous findings may reflect exercise-specific physiological demands, interindividual variability, or potential ceiling effects beyond moderate dosing. Moreover, supplementation pattern appears relevant: acute single-dose administration yielded inconsistent outcomes, whereas short-term chronic supplementation (≥7 d) produced more reliable metabolic adaptations, including increased fat oxidation and reduced respiratory exchange ratio, suggesting that repeated dosing may be necessary to optimize substrate utilization.

Anthocyanins are natural bioactive pigments belonging to the flavonoid subclass of the polyphenol family. They are widely distributed in plants and impart the red, purple, violet, and blue hues of many fruits, vegetables, and flowers. Beyond their role as natural pigments, anthocyanins found in NZBC and other dark berries are believed to influence sport performance and overall health through multiple, interconnected mechanisms [13,14,17,53]. Research has shown that anthocyanins likely enhance sport performance via improved blood flow, oxygen delivery, substrate utilization, and recovery [17,25,54]. A 7-d study indicated that although NZBC supplementation did not affect muscle performance, it did alter blood flow at rest and during prolonged submaximal isometric contractions. In terms of general health, anthocyanins contribute to cardiovascular, metabolic, immune, and cognitive protection via antioxidant and anti-inflammatory pathways [54].

In practice, moderate and long-term NZBC supplementation appears to be the most evidence-based approach for high-intensity endurance and interval training. However, heterogeneity of effects at high doses and across different sports suggests that individualized dosing may be preferable to a standardized regimen. Further large-scale trials are warranted to clarify dose–response relationships, acute compared with chronic effects, and interindividual variability in responsiveness. Overall, the quality of evidence remains low despite promising findings. Most trials were small, included recreationally active participants, and varied in exercise modality, dosing regimen, and performance measures, limiting comparability and generalizability. Additionally, within-subject variability in responses was common but often unexamined. Therefore, future research should investigate potential moderators such as sex, training status, and genetic factors affecting anthocyanin metabolism.

### Limitations

A key limitation of this review is that most of the trials included used crossover designs but did not report within-subject variability, which necessitated the use of conservative analytical assumptions that may have reduced the precision of the pooled estimates. Furthermore, the review protocol was not prospectively registered in a registry such as PROSPERO. Although the review was conducted in accordance with the PRISMA guidelines and followed a predefined methodological framework, the lack of protocol registration may limit transparency and increase the risk of methodological bias. Future systematic reviews in this area should consider prospective protocol registration to increase methodological rigor, transparency, and reproducibility.

In conclusion, NZBC supplementation may have small potential benefits for exercise performance and metabolic responses, and its effects appear to be sport-specific rather than universal. Small-to-moderate improvements have been consistently observed in high-intensity, intermittent exercise regimens, whereas evidence for endurance-based activities such as cycling and rowing remains equivocal or inconclusive. Further well-designed and adequately powered trials are needed to confirm these effects, identify responder characteristics, and develop optimal dosing strategies.

## Author contributions

The authors’ responsibilities were as follows – SG, SE: conceived and designed the study; SG, SE, AR: conducted the literature search, study selection, and data extraction; MS, ERM: contributed to data interpretation and methodological oversight; SG: drafted the initial manuscript; and all authors: critically revised the manuscript for important intellectual content, read and approved the final version of the manuscript, and take responsibility for its content.

## Data availability

All data supporting the findings of this study are included within the manuscript. Additional data are available from the corresponding author upon reasonable request.

## Funding

The authors reported no funding received for this study.

## Conflict of interest

The authors report no conflicts of interest.

## Appendix 1

### Detailed search strategy

**PubMed**

*(“Ribes nigrum”[Mesh] OR blackcurrant OR “black currant” OR cassis OR “Ribes nigrum” OR “New Zealand blackcurrant” OR “NZ blackcurrant” OR CurraNZ OR “blackcurrant extract” OR “blackcurrant supplement”) AND (“Sports”[Mesh] OR “Athletic Performance”[Mesh] OR “Exercise”[Mesh] OR sport* OR athlete∗ OR exercis∗ OR “physical activ∗” OR training) AND (“Physical Performance”[Mesh] OR “Exercise Tolerance”[Mesh] OR performance OR “exercise performance” OR “sport performance” OR endurance OR strength OR power OR sprint∗ OR fatigue OR “time trial” OR “time to exhaust∗” OR VO2 OR VO2max)∗∗. No restrictions were applied for publication date or language at the search stage.

**Embase**

*(‘Ribes nigrum’/exp OR blackcurrant OR “black currant” OR cassis OR “Ribes nigrum” OR “New Zealand blackcurrant” OR “NZ blackcurrant” OR CurraNZ OR “blackcurrant extract” OR “blackcurrant supplement*”) AND (‘sport’/exp OR ‘athlete’/exp OR ‘exercise’/exp OR sport∗ OR athlete∗ OR exercis∗ OR “physical activ∗” OR training) AND (‘physical performance’/exp OR ‘exercise performance’/exp OR ‘exercise tolerance’/exp OR performance OR “exercise performance” OR “sport performance” OR endurance OR strength OR power OR sprint∗ OR fatigue OR “time trial” OR “time to exhaust∗” OR VO2 OR VO2max)∗∗. No limits were applied for publication year or language during the search.

**SPORTDiscus (EBSCOhost)**

*(blackcurrant OR “black currant” OR cassis OR “Ribes nigrum” OR “New Zealand blackcurrant” OR “NZ blackcurrant” OR CurraNZ) AND (supplement OR extract* OR juice OR powder OR capsule∗ OR concentrate∗ OR anthocyanin∗) AND (sport∗ OR athlete∗ OR exercis∗ OR “physical activ∗” OR training OR performance OR “exercise performance” OR “sport performance” OR endurance OR strength OR power OR sprint∗ OR fatigue OR “time trial” OR “time to exhaust∗”)∗∗. No restrictions on publication date or language were applied during the search.

**Cochrane Central Register of Controlled Trials (CENTRAL)**

*(blackcurrant OR “black currant” OR cassis OR “Ribes nigrum” OR “New Zealand blackcurrant” OR “NZ blackcurrant” OR CurraNZ) AND (sport OR athlete* OR exercis∗ OR “physical activ∗” OR training) AND (performance OR “exercise performance” OR “sport performance” OR endurance OR strength OR power OR sprint∗ OR fatigue OR “time trial” OR “time to exhaust∗” OR VO2 OR VO2max) ∗∗. No limits were applied for publication date or language.
